# Conventional use and sustainable valorization of spent egg-laying hens as functional foods and biomaterials: A review

**DOI:** 10.1186/s40643-022-00529-z

**Published:** 2022-04-19

**Authors:** Hongbing Fan, Jianping Wu

**Affiliations:** Department of Agricultural, Food and Nutritional Science, 4-10 Ag/For Building, University of Alberta, Edmonton, AB T6G 2P5 Canada

**Keywords:** Spent hen, Agricultural byproduct, Sustainable utilization, Biomaterials, Functional foods, Chicken, Bioactive peptides

## Abstract

Spent hen are egg-laying hens reaching the end of their laying cycles; billions of spent hens are produced globally each year. Differences in people’s attitudes towards spent hen as foods lead to their different fates among countries. While spent hens are consumed as raw or processed meat products in Asian countries such as China, India, Korea, and Thailand, they are treated as a byproduct or waste, not a food product, in the western society; they are instead disposed by burial, incineration, composting (as fertilizers), or rendering into animal feed and pet food, which either create little market value or cause animal welfare and environmental concerns. Despite being a waste, spent hen is a rich source of animal proteins and lipids, which are suitable starting materials for developing valorized products. This review discussed the conventional uses of spent hens, including food, animal feed, pet food, and compost, and the emerging uses, including biomaterials and functional food ingredients. These recent advances enable more sustainable utilization of spent hen, contributing to alternative solutions to its disposal while yielding residual value to the egg industry. Future research will continue to focus on the conversion of spent hen biomass into value-added products.

**Graphical abstract:**

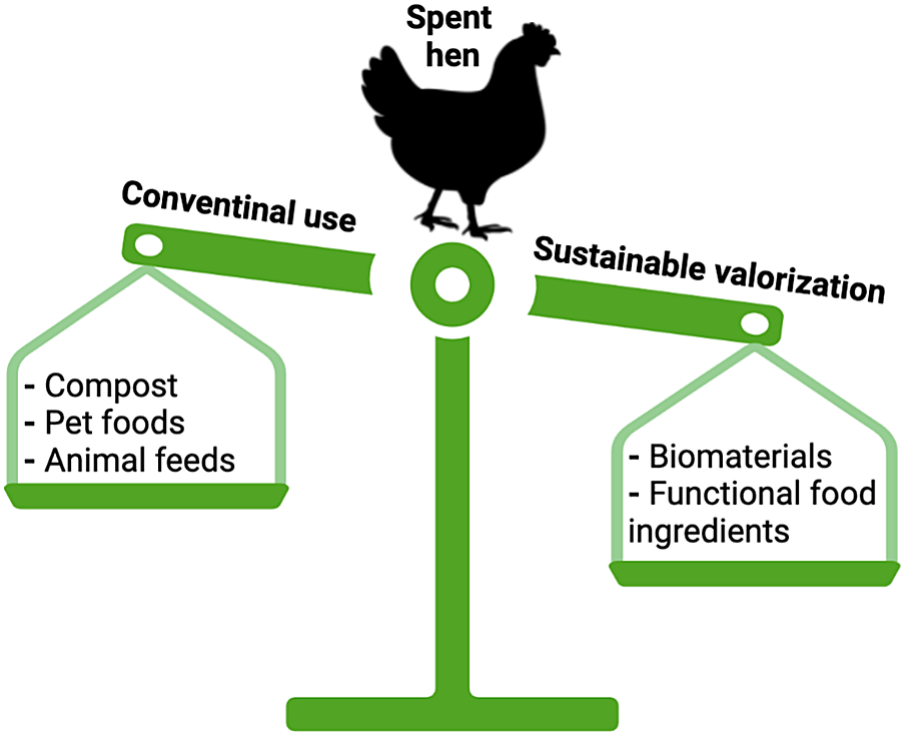

## Introduction

Chickens (*Gallus gallus domesticus*) are domesticated from red jungle fowl (*Gallus gallus gallus*). Archeological evidence demonstrated that domesticated chicken appeared about 8000 years ago in ancient China and Southeast Asia, and were subsequently spread across the globe by sailors and traders (Alders 2004; Xiang et al. 2014). Today, chickens represent by far the most important poultry species (about 90% of the poultry population), including mainly laying chicken (hens) for egg production and broilers for meat production (Alders 2004). In 2019, the global chicken population was estimated to be over 25 billion; more than 6 billion are laying hens including both in rearing and in production, contributing to an average annual egg production of more than 70 million tons over the last decade (IEC 2021; Pym 2013; Shahbandeh 2021). There is a steadily growing trend in the global egg production.

Commercial laying hens usually entails egg production for one laying cycle (~ 1 year), and then are removed from the farm due to the decline of egg-laying capacity and egg quality; these hens turn into “spent”, named spent hens. Although some hens may be extended to the second or third laying cycle, billions of spent hens are produced annually worldwide (Jacob et al. 2014; Pym 2013). Consumers’ acceptance of spent hens as foods varies among countries. In some parts of the world like China, spent hens are a regular component of table foods; they can be processed into various products such as chicken soup, snack, and processed meat product in Korea, India, and Thailand, as well as in Brazil (de Souza et al. 2011; Jin et al. 2011; Kumar et al. 2015; Sabikun et al. 2021; Sarkar et al. 2019a, b; Sorapukdee et al. 2016). In the western world, however, spent hen is generally not processed or accepted for food use, due to a low meat yield and the unacceptable toughness since its meat has a high content of collagen; the presence of brittle, and tiny bone fragments further adds cost and technical difficulty to industrial meat production. Instead, most spent hens are euthanized on the farm or in the processing plants followed by burial, composting, incineration, or rendering into oils and protein meals as animal feeds or pet foods (Cheng et al. 2004; Fritts et al. 2002; Newberry et al. 1999; Pirsich et al. 2017). Disposing spent hens by landfilling and incineration raises animal welfare and environmental concerns. Besides, farmers are liable for paying the cost for their transportation and disposal (Newberry et al. 1999). With these concerns, finding more viable, environmental-friendly approaches for spent hen disposal while yielding residue value to the egg industry are critical. Despite being treated as a waste or byproduct in some countries, spent hens are rich in animal protein and fat, which are suitable biomolecules for developing value-added products. In this review, we discussed the production, growth, and composition of spent hens, extraction of protein and fat, as well as their conventional uses (as food, animal feed, pet food, and compost) and emerging valorizations (as biomaterials and functional food ingredients), over the last several decades. Future perspectives on fully utilizing spent hens for value-added uses were also deliberated.

## Production, growth, and proximate composition

**Fig. 1:**
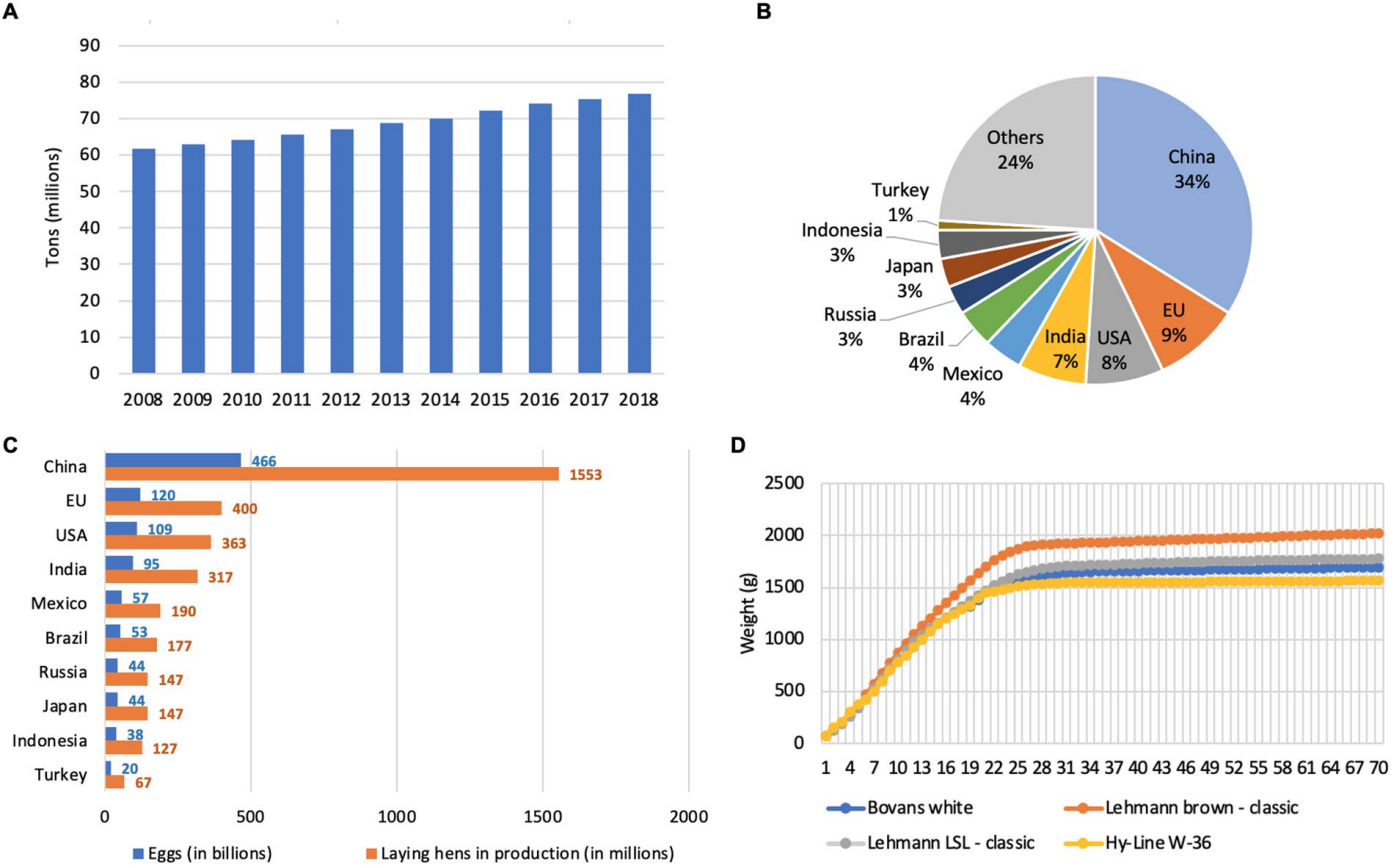
Profiles of egg and laying hen production. **A** Global egg production of 2008–2018. **B** Share of world market in egg production of global major producers in 2018. **C** Production of eggs (billions) and egg-laying hens (millions) of global major producers in 2018. Egg production was obtained from International Egg Commission; the number of spent hens were calculated based on the egg-laying capacity of 300 eggs/hen per year (Alders et al. 2018). **D** Body weight performance of several layer species. Body weight reference data of Bovans white, Lehmann brown, Lohmann Selected Leghorn (LSL), and Hy-Line W-36 referred to the management guide of New-Life Mills, Lohmann Breeders, and Ly-Line, respectively

There has been a steadily increasing trend in the global egg production since 2000 (IEC 2021). An impressive annual growth of more than 2.4% has been witnessed from 61.7 million tons in 2008 to 76.7 million tons in 2018 (Fig. 1A). In 2018, China produced 466 billion eggs, nearly one third of the world’s egg production, followed by the European Union (EU) (120 billion), USA (109 billion), and India (95 billion); the top 10 leading countries produced 1046 billion eggs, accounting for 76% of the global egg production (Fig. 1B, C). To the best of our knowledge, there is no systemic report on the population of laying hens in production. The number of global spent hens was estimated to be 4.5 billion in 2018, given that a commercial layer produces 300 eggs per year and lasts for one laying cycle (~ 1 year) (Alders et al. 2018); therefore, the estimated number of spent hens of the leading egg-producing countries is listed in Fig. 1C. However, the actual number may vary, since some layers may be extended for more laying cycles and variations in laying capacity also exist. For example, indigenous hens lay only 40–60 eggs per year while layers in the Canadian egg industry lay about 340 eggs per year (AAFC 2021; Alders et al. 2018).

A laying hen naturally produces eggs for several years, but it is common for the egg industry to keep them for only 18 months from an economic perspective. Chicks are kept in the brooder houses for 0–8 weeks, before being either transported directly to the farm or, more commonly, to a grower house, where they are reared until reaching ~ 18–20 weeks of age. Afterward they are further transported to a layer house for laying eggs, which lasts until they are about 72 weeks old (Seidler 2003). After laying eggs for nearly one year, a hen’s egg production declines to about 65% of its peak productivity, as does the egg quality (Jacob et al. 2014; Seidler 2003). In Canada and America, these hens are considered “spent” and are going to be slaughtered or euthanized on most farms (Newberry et al. 1999); in some countries however, laying hens may instead undergo a feather molt to extend the laying capacity to a second or third cycle, until observing a more significant decline in egg production (Jacob et al. 2014).

A laying hen’s body weight increases rapidly after birth until entering sexual maturity at ~ 16–24 weeks of age. Afterward, the weight still increases but at a very slow rate (Fig. 1D). Small birds, such as Bovans white and Lohmann Selected Leghorn (LSL), have an average body weight of ~ 1400 g at 20 weeks of age and ~ 1500–1800 g at 70 weeks of age; larger birds such as Lohmann brown has a higher body weight, reaching ~ 1700 g (20 weeks) and 2000 g (70 weeks) (Hy-Line 2020; Lohmann_Breeders 2019; New-Life_Mills 2016). Feather, blood, and viscera are reported to constitute about 5–7%, 2–7%, and 30% of the living body weight of chicken, respectively (Karuppannan et al. 2021; Lasekan et al. 2013). After removing feathers, hard tissues including bones and connective tissues constitute about 33% of hen carcasses weight, while the rest is soft tissues including skin, meat, viscera (Freeman et al. 2009a, b). Table 1 lists the proximate composition of spent hens from our recent analysis and also several published studies. Overall, spent hen carcass has a high content of protein; it is also high in fat but its content is low in meat, which indicates the presence of fat in other tissues such as viscera as well as the high content of abdominal and subcutaneous fats (Peña-Saldarriaga et al. 2020).

**Table 1:** Proximate composition, fatty acid and amino acid profiles of raw spent hen and meat

Sample	Moisture (%)	Protein (%)	Fat (%)	Ash (%)	Fatty acid profile	Major amino acid	References
Spent hen carcass^a^	59.8	18.5	14.9	5.3			
Whole spent hen^b^	58.2	17.9	19.1	4.5		Glu, Gly, Asp, Leu, Arg, Pro, Lys	Freeman et al. (2009a, b)
Whole spent hen^c^	67.7	17.1	14.1	2.1			Zubair (2017)
Whole spent hen^d^	60.1	21.4	16.3	2.2	SFA (28%), MUFA (50%), PUFA (22%)		Safder et al. (2019a, b)
Spent hen breast meat	72.0	22.9	1.5	1.5		Glu, Leu, Arg, Lys, Asp	Okarini et al. (2013)
Spent hen breast meat	73.9	21.7	3.8	1.3	SFA (38%), MUFA (33%), PUFA (29%)		Semwogerere et al. (2019)

*SFA* saturated fatty acids, *MUFA* mono-unsaturated fatty acids, *PUFA* poly-unsaturated fatty acids

^a^Spent hen carcass (without feather, head, feet, and viscera), analyzed by our lab

^b^Whole spent hen (with feather)

^c^Whole spent hen (including skin, bone, muscle, leg, and gizzard)

^d^Whole spent hen (no indication on body components)

## Conventional uses of spent hens

### Foods

In China, spent hens are popular table dishes such as stewed or braised chicken in both home kitchens and restaurants. In other Asian countries, particularly in India, spent hens are further processed into various products or snacks, such as sausage, cutlet, jerky, kachori, nugget, patty, and tikka, among others (de Souza et al. 2011; Kumar and Sharma 2006; Kumar et al. 2015; Rajeshwar et al. 2018; Sabikun et al. 2021; Singh et al. 2001, 2015; Sorapukdee et al. 2016). Due to the objectionable toughness of spent hen meat, additional tenderization has been studied. A number of studies reported the use of natural tenderizers in improving texture, tenderness, and sensory attributes of spent hen meat, such as ginger extract, papaya leaves, pineapple rind powder, and kiwifruit proteases, despite with a moderate success (Abdalla et al. 2013; Bhaskar et al. 2006; Kantale et al. 2019; Sangtherapitikul 2004; Sharma and Vaidya 2018). Besides, spent hen meat is also an ingredient for surimi products (Jin et al. 2007; Nowsad et al. 2000). Spent hen surimi has been reported to possess better thermal gelation property than that of broiler surimi, but its gel quality is deteriorated at a faster rate over storage. Thus, cryoprotectants should be applied to retard the deterioration of gel characteristics of spent hen surimi, such as sucrose, sorbitol, phosphate, and so on (Jin et al. 2011; Wang et al. 2013). Other than as table dishes or as the major ingredient of a meat product, spent hen meat can also be incorporated as a minor component into other food products. Umaraw and Chauhan (2018) reported that substitution of 30% whole wheat flour with spent hen meat powder in bread maintained sensory acceptability without compromising product quality. Lee et al. (2003) demonstrated that incorporating spent hen meat as ratios of 1:2 and 1:3 (meat to corn or potato starches) into the formulation of popped cereal snacks did not impair the required product characteristics. Li (2006) reported that adding 6% of spent hen myofibrillar proteins increased functional properties such as gumminess and chewiness of chicken breast or pork ham.

Homemade soup is a time-consuming process, thus spent hens have also been processed into instant soup. The addition of 25% of spent hen meat shred in an instant soup mix provided extra nutrition without impairing the sensory qualities; the soup could be aerobically stored at ambient temperature for a period of up to 90 days (Sarkar et al. 2019a, b). Certain treatments such as shredding, pressure cooking, flavoring, and adding thickening agents can further improve the product quality (Sarkar et al. 2019a, b). Indeed, chicken soup has long been considered as a healthy food which has medicinal effects, being traditionally used to treat colds and upper respiratory infections (Caroline and Schwartz 1975; Lipman 2003; Rennard et al. 2000; Saketkhoo et al. 1978). Recently, some researchers recommended homemade chicken soup as a potential folk remedy to boost immune function against the coronavirus disease 2019 (Rennard et al. 2020). These evidences demonstrated the health-beneficial nature of spent hens as foods.

### Animal feeds and pet foods

Many spent hens are sent for rendering into oils or protein meals as animal feed or pet food. Other than being slaughtered and cut into pieces, whole spent hens are more feasible from an economic perspective (Karthik et al. 2010; Pirsich et al. 2017). For example, rendered whole spent hen meal was acceptable as a protein and nutrient source for commercial broiler from hatch to 6 weeks of age; replacing up to 12% of broilers diet with rendered spent hen meal did not cause sensory dissatisfaction on meat quality (Christmas et al. 1996; Williams and Damron 1998). Incorporating 5–10% of spent hen meal into a regular corn basal diet improved early postmolt performance of laying hens, without altering the egg quality and acceptability significantly (Koelkebeck et al. 2001). Besides, a spent hen hard tissue meal made by feathers, bones, and connective tissues maintained nitrogen metabolism in goats, similarly to that of traditional protein sources (Freeman et al. 2009a, b). More trials of utilizing spent hen meal for poultry, livestock, ruminant, and aquaculture feedings with improved both animal nutrition and end product quality have been reported (Bravo Jimenez et al. 2009; Cheng et al. 2004; Douglas and Parsons 1999; Rojas and Stein 2013; Williams and Damron 1999).

Feed Ban Acts have been implemented for ruminant feed in many countries since 1990s, due to the concern towards the occurrence of animal diseases such as bovine spongiform encephalopathy. In North America, the ban mostly applies to mammalian-derived protein meals, not including poultry proteins (CFIA 2015; Regulations 2016), which may significantly impact the use of poultry meal in livestock feed. As comparison, Australia has introduced a more stringent feed ban since 1996 and all animal meals have been prohibited in ruminant feed, except for milk, gelatin and tallow (Australia 2021). In 1994, European Union issued a ban of feeding mammalian-derived proteins to ruminants; the ban has been extended to all processed animal meals including all farmed animals in 2001. Although the ban was partially lifted in early 2021, which allowed poultry meals to be used in pig feed, a lot has changed since animal meals were first banned 2 decades ago (Commission 2021). Regulations restricting rendering poultry byproducts into animal feeds speeds up the development of new applications for spent hens.

Poultry protein meal makes up an important share of premium pet foods, possessing good palatability and meeting well the nutritional requirements for amino acids and fatty acids (Aldrich 2006). The soft tissues of spent hens, including striated muscle, viscera, and other organ tissues, have high protein and low ash contents and are thought a good option (Aldrich 2006; Krestel-Rickert 2001). As reported, incorporating 10% of whole spent hen meal into dog foods provides good nutritive value while also maintains product quality and shelf life (Karthik et al. 2010). Note that rendered spent hen meal has a high content of fat which is rich in highly unsaturated fatty acids (e.g., oleic acid, linoleic acid), thus antioxidants are suggested to be used over a longer period of storage (Fritts et al. 2002; Safder et al. 2019a; Semwogerere et al. 2019).

### Compost

Composting decomposes organic substances for agricultural soil amendment, enriching the soil with nitrogen, phosphorus, and potassium, and other nutrients that are essential for plant growth. Since 1980s in the USA, composting poultry waste such as feathers and spent hen carcasses (often with carbon sources such as straw and woodchip) have been implemented widely by poultry producers, being more advantageous than burial and incineration that create groundwater and air quality issues (Malone 2004; Newberry et al. 1999). Compared with manure, composting experiences less foul smell and nutrient leaching as well as introduces less or no pathogenic microorganisms such as *E. coli* and *salmonella* (Laca et al. 2021). Not many reports are available on the effect of spent hen compost on soil amendment or growing crops in the literature; however, temperature, moisture, carbon and nitrogen contents need to be monitored, as well as the emissions such as ammonia, carbon dioxide, methane, nitrogen oxide and dioxide (Spencer 2011). Kucinska et al. (2014) examined the effect of hen feather compost using *Bacillus polymyxa* B20 on the growth characteristics of several plants (cucumber, cabbage, tomato, and maize), including fresh weight, leaf chlorophyll content, and activity of several regulatory enzymes. The composting process released amino acids from feather (containing mainly keratin) which significantly promoted plant growth. Many other fungi such as *Chrysosporium europae* and *Microsporum gypseum* have been used to degrade feather keratin and release amino acids (Parihar and Kushwaha 2000).

## Extraction of crude proteins and fat from spent hens

Protein and fat need to be extracted from spent hens before being developed as value-added products. Fat can be extracted using organic solvents such as chloroform and methanol mix (2:1, *v/v*) (Folch method), followed by evaporation of the lower layer; more than 95% of fat could be recovered within 10 min with the assistance of microwave technology (Safder et al. 2019a). Besides, Safder et al. (2019b) adopted supercritical carbon dioxide to extract fat from spent hens, with satisfactory extraction yield of 37% (dry basis) and recovery of 91.4% at 50 MPa/70 °C. Fat derived from whole spent hen consists of ~ 28% saturated fatty acids (SFA), 50% mono-unsaturated fatty acids (MUFA), and 22% poly-unsaturated fatty acids (PUFA), respectively, while that from breast meat contains 38% SFA, 33% MUFA, and 29% PUFA, respectively; SFA, MUFA, and PUFA are dominated by palmitic acid, oleic acid, and linoleic acid, respectively (Safder et al. 2019a; Semwogerere et al. 2019). Peña-Saldarriaga et al. (2020) also reported similar proportions of SFA (30%), MUFA (41%), and PUFA (24%) in chicken gizzard and abdominal fats, as well as the dominant fatty acids in each category.

**Fig. 2:**
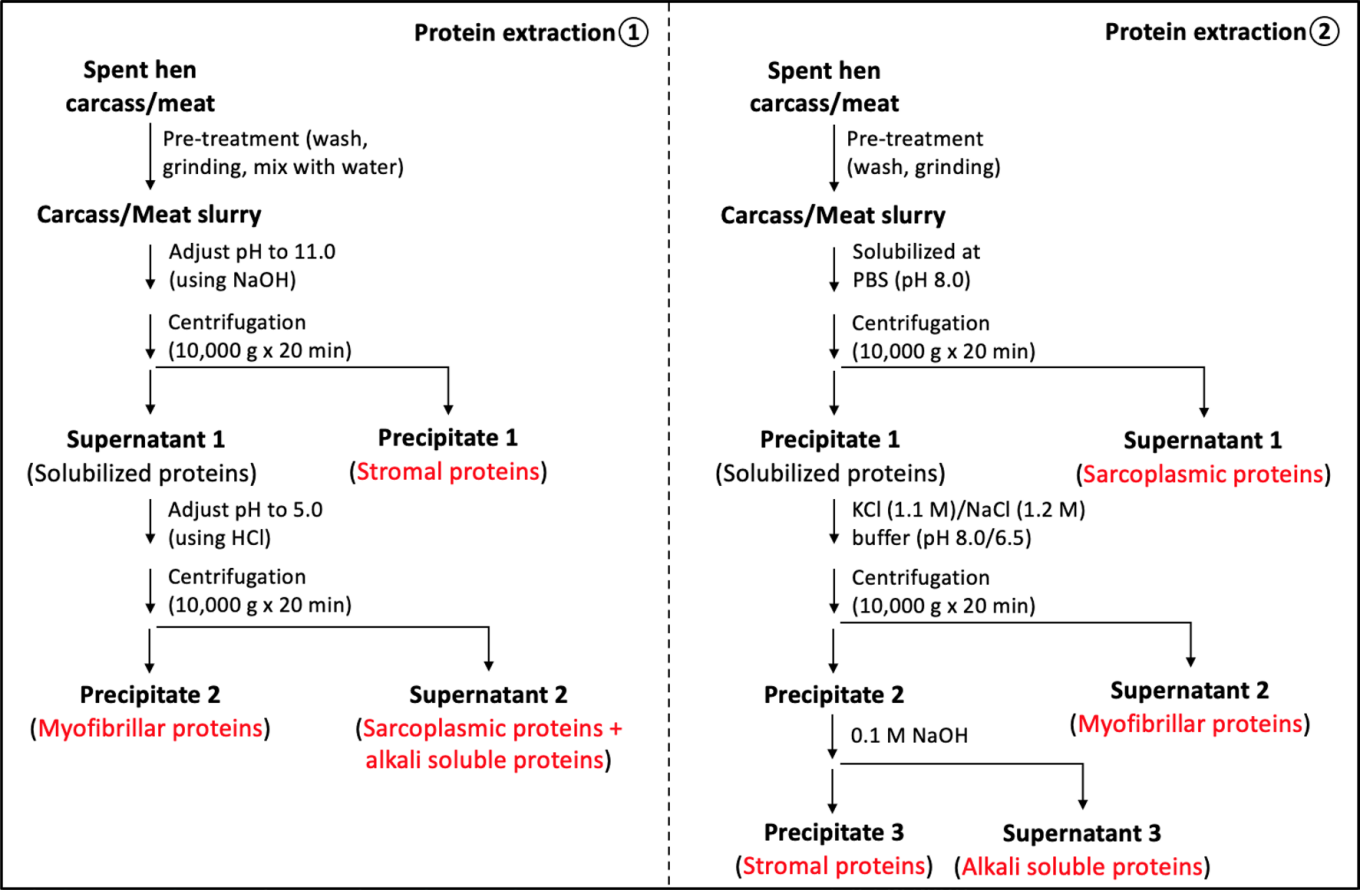
Protein extraction from spent hens. Protein extraction protocol 1 was summarized from Wang and Wu (2012) and Fan et al. (2021) with slight modifications; protein extraction protocol 2 was summarized from Zubair (2017) with slight modifications. Supernatants can be freeze-dried to obtain proteins, or be further dialyzed before collecting the retainates for freeze-drying

Myofibrillar proteins and collagen are the dominant proteins in spent hen carcasses. There are two primary protein extraction methods, whose principles are both based upon the differences in solubility of proteins at certain pHs (Fig. 2). Wang and Wu (2012) solubilized spent hen meat proteins at alkaline pH (11.0) and subsequently separated myofibrillar proteins through precipitating the supernatant at its isoelectric point (pH 5.0). Other researchers used similar techniques (Fan and Wu 2021b; Fan et al. 2020; Udenigwe et al. 2017; Yu et al. 2018a). A high purity of protein sample (~ 93.2%) could be obtained using this pH-shift method (Fan et al. 2020). Meanwhile, using this method, other proteins, such as sarcoplasmic proteins and stromal proteins (e.g., collagen and elastin), can also be fractionated at different pHs. Unlike separating proteins by precipitation, Zubair (2017) solubilized proteins and separated supernatants step by step at different pHs. Sarcoplasmic proteins were first enriched in the supernatant of spent hen slurry and further fractionated by acetone precipitation. Afterward myofibrillar proteins in the precipitate were solubilized by adding high content of salt such as KCl (1.1 M) or NaCl (1.2 M), from which the second supernatant was dialyzed, with the retentate collected as myofibrillar proteins; the resultant precipitate was further solubilized in NaOH to obtain stromal proteins (Fig. 2). Under the optimized technological parameters, 74% of proteins were recovered with a purity of 96% (Zubair 2017). Overall, the above two methods yield protein extracts with similar recovery and purity, but lack of studies in comparing physiochemical properties and quality of the extracted proteins makes it difficult to specify their comparative advantages and applications at this moment. With regard to the amino acid composition, spent hen meat is rich in glutamic acid, leucine, arginine, lysine, and aspartic acid (Table 1) (Okarini et al. 2013; Sangtherapitikul 2004); whole spent hen carcass is also rich in glycine and proline, which is possibly due to the high content of collagen (Freeman et al. 2009a, b).

## Value-added uses of spent hens

Research on developing spent hen for value-added applications is still in its infancy, mainly focusing on proteins and lipids, which dominate spent hen dry matter (Table 1). Many trials have been conducted or are ongoing to transform spent hen proteins into bioactive peptides with health-beneficial effects, mainly muscle proteins and collagens (Table 2) (Fan et al. 2021; Fan and Wu 2021b; Hong et al. 2021; Offengenden et al. 2018). Besides, much endeavor has also been put into developing protein or lipid-based biomaterials as potential substitutes for synthetic materials (Table 3) (Pradhan et al. 2020; Safder et al. 2019b, 2020; Wang and Wu 2012). Developing more value-added uses of spent hens align with the global trend of valorization of agricultural byproducts (McHugh 2019).

**Table 2:** Bioactivities or functionalities of spent hen proteins (or derived peptides)

Proteins (tissues)	Bioactive peptides (functionalities)	Processing conditions for preparing hydrolysates or peptides^a^	Product characterization	References
Raw meat	Antioxidant peptides	- Prepared by flavourzyme or alcalase simultaneously or sequentially (E/S 1–3%, 50–55 °C, pH 6.5–7.5 for up to 6 h)	- The hydrolysate after a sequential hydrolysis using alcalase and flavourzyme showed higher degree of hydrolysis - Flavourzyme-digested hydrolysate showed higher protein recovery and antioxidant activity (DPPH-scavenging activity, FRAP, and FICA)	Kumar et al. (2020)
Raw meat	Antioxidant activity, bioaccessibility, and solubility	- Prepared by Flavourzyme (E/S 3%, pH 6.6, 54 °C, 30 min) - Dried either spray-drying (SD) or freeze-drying (FD)	- FRAP: SD > FD - DPPH-scavenging activity: SD > FD - Particle size: SD < FD - Flowability: SD > FD - Bioaccessibility: SD > FD - Protein content SD < FD - Solubility: SD < FD	Kumar et al. (2021)
Raw meat	Antioxidant and ACEi activities	- Prepared by alcalase (55 °C, pH 7.0), flavourzyme (50 °C, pH 7.0), neutrase (50 °C, pH 6.0), protamex (40 °C, pH 7.0), pepsin (37 °C, pH 3.0) and trypsin (37 °C, pH 8.0) for up to 6 h (E/S 1%) - Added hydrolysates into crab meat analogue	- Incorporation of 1.5% of the hydrolysate increased DPPH- and hydroxyl radical-scavenging activities of crab meat analogue - Incorporation of 1.0% of the hydrolysate increased ACEi activity of crab meat analogue	Jin et al. (2016)
Raw meat	Antioxidant activity	- Prepared by Protamex (E/S 5%, 43 °C, pH 7.0) for 1 h followed by Bromelain (E/S 1%, 50 °C, pH 7.0) - Added 1% or 4% hydrolysate powder (dry basis) into boiled fish paste followed by storage at 10 °C over 4 weeks	- Antioxidant activity (DPPH-scavenging activity) of boiled fish paste was increased - Physicochemical and sensory properties were reduced	Hur et al. (2016)
Muscle proteins	Renin, ACEi, antioxidant, and antihypertensive activities	- Prepared by pepsin (E/S 1%, pH 2.0) at 37 °C for 1.5 h - Prepared by pepsin (E/S 1%, pH 2.0, 1.5 h) and pancreatin (E/S 1%, pH 7.5, 3 h) at 37 °C sequentially	- Renin inhibition (IC_50_ value: 0.34–0.52 mg/mL) - ACE inhibition (IC_50_ value: 0.42–0.65 mg/mL) - Bovine plasma oxidation-inhibitory activity (plasma sulfhydryl content and FRAP) - Reduced systolic blood pressure by 26.5 and 36.8 mmHg in spontaneously hypertensive rats	Udenigwe et al. (2017)
Muscle proteins	Antioxidant, ACEi, and anti-inflammatory peptides (IWHHT, IWH, IW)	- IWHHT was prepared by thermolysin (E/S 0.5%, 60 °C, pH 8 for 3 h) - IWH and IW were gastrointestinal digests of IWHHT	- IWHHT/IW had ACE IC_50_ values of 9.93/2.0 µM - IWHHT, IWH, and IW reduced basal oxidative stress in endothelial cells (DHE staining assay) - IWHHT and IWH attenuated TNFα-induced inflammation (reduced VCAM-1 expression by 40–60%) in endothelial cells - IWHHT, IWH, and IW were transported intact in Caco-2 cell monolayers	Fan et al. (2018), Gu et al. (2019)
Muscle proteins	Anti-inflammatory peptides (WPW, FLWGKSY, AGLLGLL, SFMNVKHWPW, AFMNVKHWPW, TFLPMLQHIS, ASLSTFQQMWITK	- Prepared by Protex 50FP (E/S 4%, 50 °C, pH 3.0 for 3 h)	- The hydrolysate increased interleukin-10 level in Sprague-Dawley rats - The hydrolysate and derived peptides showed in vitro interleukin-6 inhibitory activity in endotoxin-activated macrophage-like U937 cells	Yu et al. (2018a, b)
Muscle proteins	Antioxidant, anti-inflammatory, ACEi, and ACE2u activities	- Prepared by 9 enzymes (E/S 4%, 3 h) individually or in combination, including alcalase (pH 8, 50 °C), Protex 6L (pH 8, 37 °C), Protease S (pH 8, 37 °C), thermoase (pH 8, 60 °C), trypsin (pH 8, 60 °C), protease M (pH 8, 60 °C), pepsin (pH 2, 60 °C), Protex 50FP (pH 3, 60 °C), and Protex 26L (pH 3, 60 °C)	- 18 hydrolysates were prepared; 3 hydrolysates prepared by Protex 26L, pepsin, or thermoase showed high multifunctional bioactivities and peptide yield - Thermoase-digested hydrolysate maintained its bioactivities after gastrointestinal digestion and transport across Caco-2 cells - Thermoase-digested hydrolysate reduced blood pressure in spontaneously hypertensive rats in a preliminary trial	Fan et al. (2020)
Muscle proteins	Blood pressure reduction	- Prepared by thermoase (pH 8, 60 °C, 3 h) - The hydrolysate was orally administrated at 1 g/kg body weight to spontaneously hypertensive rats, with blood pressure monitor 24 h per day every 2 days rover 20 days	- Thermoase-digested hydrolysate reduced systolic blood pressure from 168.7 to 156.8 mmHg - Modulated the renin–angiotensin system components (increased plasma and vascular levels of ACE2 and angiotensin (1-7); reduced plasma angiotensin II concentrations - Attenuated vascular inflammation, oxidative stress and fibrosis	Fan and Wu (2020), Fan et al. (2022)
Muscle proteins	ACEi, ACE2u, and antioxidant peptides	- Prepared by thermoase (E/S 4%, pH 8, 60 °C, 3 h)	- 5 ACEi peptides (IC_50_ values of 0.034–5.77 μg/mL): VRP, LKY, VRY, KYKA, and LKYKA - 4 ACE2u peptides (increased vascular ACE2 expression by 0.52–0.84 folds): VKW, VHPKESF, VVHPKESF and VAQWRTKYETDAIQRTEELEEAKKK - 4 peptides (VRP, LKY, VRY, and VVHPKESF) showed antioxidant activity in vascular cells	Fan and Wu (2021a, b, c); Fan et al. (2021)
Muscle proteins	Human bitter taste receptor-blockers	- Prepared by Protease S, alcalase, Protex 6L, and Protex 50FP were assessed on their bitter taste receptor-blockers by electronic tongue and also in HEK293T cells	- The Protex 50FP-digested hydrolysate has the lowest bitterness - A number of peptides identified from Protex 50FP-digested hydrolysate inhibited quinine- and diphenhydramine-mediated bitter sensation	Xu et al. (2019)
Elastin (Skin)	Antioxidant peptides	- Prepared by alcalase (pH 8.5, 60 °C) and elastase (pH 8.5, 37 °C) for 2, 4, 8, 12, 16 or 24 h	- DPPH-scavenging activity (16–50%) - ABTS-scavenging activity (60–79%) - Fe^2+^ chelating activities (50–77%)	Nadalian et al. (2015)
Elastin (Skin)	ACEi activity	- Prepared by alcalase (pH 8.5, 60 °C) and elastase (pH 8.5, 37 °C) for 2, 4, 8, 12, 16 or 24 h	- Both elastin hydrolysates and its fraction (< 3 kDa) exhibited ACEi activity	Yusop et al. (2016)
Collagen (from meat)	Antioxidant, anti-inflammatory, proliferative, and type I collagen synthetic activities	- Prepared by protease M (pH 3.0), alcalase (pH 8.0), Protex 50FP (pH 3.0), Protex 51FP (pH 7.5), by an individual enzyme (2 h) or in combination (2 h for each enzyme) (E/S 2%, 50 °C) (10 hydrolysates)	In TNFα-stimulated human dermal fibroblasts - Five hydrolysates reduced oxidative stress - Six hydrolysates reduced inflammation (inhibited ICAM-1 and VCAM-1 expressions) - Two hydrolysates promoted cellular proliferation - One hydrolysate increased type I procollagen synthesis	Offengenden et al. (2018)
Collagen (Skin)	LWM peptides	- Pepsin (E/S 1%, pH 2.0) for 24 h of pretreatment followed by hydrolysis by papain (E/S 2%, pH 6.0, 60 °C, 6 h)	- Pepsin treatment enhanced production of LMW collagen peptides (to 32.59%) by removing telopeptides and reduces cross-links	Hong et al. (2017)
Collagen (Skin)	LMW peptides	- Formic acid treatment of pepsin- (E/S 1%, pH 2.0, 24 h) or heat-soluble collagens, before hydrolysis by papain (E/S 2%, pH 6.0, 60 °C, 6 h)	- Formic acid treatment enhanced production of LMW collagen peptides (from 36.32 to 43.34%) for pepsin-soluble or (33.79–48.92%) for heat-soluble collagen by removing telopeptides and reducing cross-links	Hong et al. (2018)
Collagen (Skin)	LMW peptides	- α-amylase pretreatment (E/S 2%, pH 5.4, 20 °C, 6 h) followed by hydrolysis by papain (E/S 5%, pH 6.0, 60 °C, 6 h)	- α-amylase pretreatment improved LMW peptide (< 2 kDa) yield from 33.79 to 67.66%	Hong et al. (2021)
Collagen (Skin)	Anti-aging of LWM peptides	- Produced by papain hydrolysis after formic acid and pepsin pretreatments (Hong et al. 2018)	In human dermal fibroblasts with ultraviolet A-exposure after treatment with the hydrolysate of 1 mg/mL - Increased cell viability (by 1.7 folds) - Reduced ROS generation (by 26%) - Increased type I α-procollagen production (by 1.5 folds) - Reduced MMP-1 (by 27) and MMP-9 (by 67%) synthesis - Reduced apoptotic genes (Bax and caspase-9)	Wang et al. (2019a, b)

^a^Processing condition includes enzymatical hydrolysis parameters (enzyme/substrate (E/S), temperature (T), and pH value)

ABTS: 2,2ʹ-azino-bis(3-ethylbenzothiazoline-6-sulfonic acid); ACEi: angiotensin-converting enzyme (ACE) inhibitory; ACE2u, angiotensin-converting enzyme 2 (ACE2u) upregulating; DHE: dihydroethidium; DPPH: 2,2-diphenyl-1-picrylhydrazyl; FICA: ferrous ion chelating activity; FRAP: ferric reducing antioxidant power; ICAM-1: intracellular adhesion molecule-1; MMP: metalloprotease; LMW: low-molecular-weight; TNFα: tumor necrosis factor alpha; VCAM-1: vascular cell adhesion molecule-1

### Functional foods

Bioactive peptides have been an emerging functional food ingredient due to their excellent health benefits in preventing or treating various diseases (Esfandi et al. 2019; Fan and Wu 2021a; Hong et al. 2019; Lammi et al. 2019; Wang et al. 2019b). Animal proteins such as myofibrillar proteins and collagen represent excellent sources of bioactive peptides (Hong et al. 2019; Toldrá et al. 2018; Udenigwe and Howard 2013). Overall, spent hen proteins can be easily transformed into health-promoting bioactive peptides primarily through enzymatic hydrolysis, but the yield and recovery of peptides heavily depends on the enzymes used (Fan et al. 2020; Offengenden et al. 2018). For example, different proteases possessed varying efficiencies in releasing bioactive peptides from spent hen muscle proteins, with peptide yield of 44.4–86.5% (Fan et al. 2020). As shown in Table 2, bioactive peptides with various properties have been developed from spent hen proteins, such as renin-inhibitory, angiotensin-converting enzyme (ACE) inhibitory (ACEi), ACE2 upregulating (ACE2u), antioxidant, anti-inflammatory, and anti-aging activities, among others. Their bioactivities are evaluated using in vitro or in vivo assays, either alone or in the form of protein hydrolysates.

#### Raw meat (antioxidant and ACEi peptides)

Kumar et al. (2020) prepared several spent hen meat protein hydrolysates using flavourzyme and alcalase individually, simultaneously, or sequentially. Overall, flavourzyme-digested hydrolysate had higher protein recovery and antioxidant potency than those of the other hydrolysates, albeit with a lower degree of hydrolysis. Subsequently, the same researchers further compared the effect of different drying methods (spray-drying vs. freeze-drying) on antioxidant activity, bioaccessibility, and product characteristics of the flavourzyme-digested hydrolysate (Kumar et al. 2021). Spray-drying rendered the hydrolysate higher antioxidant activity, bioaccessibility, and flowability but a smaller particle size and lower solubility and protein content, than freeze-drying. Taken together, the researchers concluded that spent hen meat hydrolysate powders obtained by spray- and freeze-drying were promising functional food ingredients or nutraceuticals.

#### Myofibrillar proteins (antioxidant, anti-inflammatory, antihypertensive, renin, ACEi, and ACE2u peptides)

Myofibrillar proteins are the most abundant proteins in meat. Udenigwe et al. (2017) extracted myofibrillar proteins from spent hens and prepared two hydrolysates using pepsin alone or pepsin and pancreatin; both hydrolysates inhibited in vitro renin and ACE activities, reduced plasma oxidation, and lowered blood pressure significantly in spontaneously hypertensive rats. Gu et al. (2019) prepared spent hen muscle protein hydrolysate using thermolysin (ACEi IC_50_ value of 39.6 µg/mL) and identified an actin-derived ACEi peptide, IWHHT, with great antioxidant, anti-inflammatory, and antihypertensive activities. Further studies revealed that IWHHT could be further digested by gastrointestinal proteases into two fragmentary peptides, IWH and IW; both peptides exhibited similar or enhanced ACEi, antioxidant, anti-inflammatory, or antihypertensive property compared with those of IWHHT (Fujita et al. 2000; Gu et al. 2019). All three peptides can be transported intact across Caco-2 monolayer (Fan et al. 2018). A spent hen muscle protein hydrolysate prepared by thermoase PC10F, a food-grade protease, could significantly reduce blood pressure in spontaneously hypertensive rats over a period of 20 days (Fan et al. 2020). Associated with the blood pressure reduction were increased plasma ACE2 level, increased vascular expression of ACE2, as well as attenuated inflammation, oxidative stress, and fibrosis (Fan et al. 2022; Fan and Wu 2020). Later, several potent ACEi peptides including Val-Arg-Pro (VRP), Leu-Lys-Tyr (LKY), and Val-Arg-Tyr (VRY), with IC_50_ value of 0.034–5.77 μg/mL were identified, as well as a few ACE2u peptides, such as Val-Lys-Trp (VKW), Val-His-Pro-Lys-Glu-Ser-Phe (VHPKESF), and Val-Val-His-Pro-Lys-Glu-Ser-Phe (VVHPKESF), which upregulated vascular ACE2 expression by 0.52–0.84 folds (Fan and Wu 2021b). Among these peptides, VRP, LKY, VRY, and VVHPKESF showed great antioxidant effects in vascular endothelial and smooth muscle cells (Fan et al. 2021); VVHPKESF also reduced blood pressure in spontaneously hypertensive rats (Fan and Wu 2021c). In addition, a spent hen muscle protein hydrolysate prepared by Protex 50FP exhibited in vitro inhibitory activity of interleukin (IL)-6, a pro-inflammatory cytokine; seven IL-6 inhibitory peptides have been identified (Yu et al. 2018a). However, in a subsequent 3-week feeding trial in Sprague-Dawley rats, inhibition of IL-6 in vivo has not been witnessed, but the production of IL-10, an anti-inflammatory cytokine (Yu et al. 2018b).

#### Stromal proteins (antioxidant, anti-inflammatory, anti-aging, and ACEi peptides)

Stromal proteins are also known as connective tissue proteins, including collagen, elastin, and reticulin. Collagen is the most abundant protein in the animal kingdom, present in various fibrous tissues such as skin, bone, tendons, and muscle. Collagen peptides have been reported with various beneficial effects including cardiovascular protection, joint pain relief, skin and bone health, and so on (Hong et al. 2019). However, production of collagen peptides from terrestrial animal sources (e.g., bovine, porcine, and chicken) confronts big technical challenges due to the nature of high crosslinking, which represents the major barriers of enzymatic degradation of collage fibers (Hong et al. 2019). Recently, an array of technologies have been used to prepare low-molecular-weight (LMW) collagen peptides from spent hens with great success (Table 2). Pretreatment with pepsin removed the crosslinked telopeptides in collagen molecules, improving substantially spent hen skin collagen proteolysis by papain and thus a higher LMW peptide yield (Hong et al. 2017). Next, treatment with formic acid post pepsin pretreatment further enhanced collagen proteolysis by papain, since formic acid removes collagen cross-links; formic acid treatment also enhanced proteolysis of heat-soluble collagen (Hong et al. 2018). More recently, Hong et al. (2021) reported that pretreatment with α-amylase, which destructs advanced glycation end products (AGEs) cross-links, increased LMW peptide (< 2 kDa) yield (from 33.8 to 67.7%) from spent hen skin collagen. A combination of these techniques may work synergistically and further enhance LMW peptide production, therefore further investigations are warranted.

Spent hen collagen peptides have been evaluated for their health-promoting benefits. A skin collagen hydrolysate prepared by Hong et al. (2017, 2018) demonstrated anti-aging activity in human dermal fibroblasts post ultraviolet A-exposure (Wang et al. 2019a). Pretreatment with 1 mg/mL of the hydrolysate significantly enhanced cell viability (by 1.7 folds) and type I procollagen level (by 1.5 folds), reduced oxidative stress (by 26%), inhibited metalloproteinase (MMP)-1 (by 27%) and MMP-9 (by 67%) synthesis, and downregulated apoptotic genes including Bax (by 29%) and caspase-9 (by 61%); these effects were possibly mediated via discoidin domain receptor 2 followed by Akt and extracellular signal-regulated kinase 1/2 signaling pathways (Wang et al. 2019a). Collagen peptides derived from spent hen meat also improved skin health, exhibiting great antioxidant, anti-inflammatory, proliferative, and type I procollagen-synthetic activities in human dermal fibroblasts (Offengenden et al. 2018).

Elastin is another type of stromal proteins. Yusop et al. (2016) extracted water-soluble elastin from spent hen skin and found that elastin hydrolysates prepared by alcalase and elastase possessed great antioxidant activities. Likewise, Nadalian et al. (2015) analyzed the antihypertensive potential of these elastin hydrolysates; both hydrolysates and their fractions (< 3 kDa) possessed great in vitro ACEi activities.

#### Spent hen-derived peptides as functional ingredients into food products

Spent hen meat derived peptides have been incorporated into food system for practical application. For example, Jin et al. (2016) developed a spent hen meat hydrolysate using six enzymes (alcalase, flavourzyme, neutrase, protamex, pepsin, and trypsin); incorporation of the hydrolysate into crab meat analogue resulted in significantly enhanced antioxidant and ACEi activities over 6 weeks of storage. Hur et al. (2016) studied the effect of incorporating a deboned spent hen meat hydrolysate, prepared by protamex and bromelain, on antioxidant characteristics of boiled fish paste. Antioxidant activity of the fish paste was increased, but its physicochemical and sensory properties were slightly reduced.

We have previously reported that spent hen muscle protein hydrolysate prepared by Protex 50FP generated a hydrophilic fraction, which significantly reduced bitter sensation of quinine and attenuated the activation of bitter taste receptors in HEK 293 cells (Xu et al. 2019). Another muscle protein hydrolysate prepared by food-grade protease thermoase PC10F possessed weak bitterness but strong umami taste (Fan et al. 2022). This indicated the applicability of spent hen peptides in food matrices as flavor enhancers or off-flavor masking agents. Spent hen peptides have a theoretical base to be of good flavor, due to the abundancy of glutamic acid, a big contributor to umami taste (Maehashi et al. 1999) (Table 1). It is warranted to optimize the hydrolysis conditions to prepare spent hen hydrolysates or peptides with health benefits and pleasant flavor.

### Biomaterials

The overwhelming use of synthetic materials raises concerns about environmental security and sustainability. Given the raw materials of synthetic materials excessively rely on fossil resources, which is vulnerable to changes in global policies and politics, biobased materials have attracted considerable interest from researchers and industrial observers in recent years. Obvious advantages using biomolecules-based biomaterials include their biodegradability, cost-effectiveness, and wide availability. Recent studies on developing spent hen protein- or lipid-based biomaterials are shown in Table 3.

**Table 3:** Biomaterial applications of spent hen proteins and lipids

Proteins/lipids	Application (Products)	Processing conditions for preparing biomaterials	Product characterization	References
Muscle proteins	Wood adhesive	- Modified by 3% sodium dodecyl sulfate or 3 M urea	- The modification promoted protein unfolding, exposing more secondary structures that strengthen protein-wood bonding - Interaction between proteins and modification agents enhanced mechanical interlocking - The prepared adhesives can be applicable in both dry and wet environments	Wang and Wu (2012)
Muscle proteins	Bionanocomposite film	- Compression molding using glycerol (plasticizer), chitosan (cross-linker), and nanoclay (nanoparticles)	- The exfoliated bionanocomposite film had improved thermal, thermomechanical, and barrier properties, with possible uses as food packaging materials	Zubair et al. (2019)
Collagen (Gelatin)	Hydrogel	- Gelatin was made from extracted spent hen collagen - Gelatin scaffold was formed through crosslinking by adding glutaraldehyde	- Properties of gelatin scaffold: porosity (90%), pore size (range of 104–244 µm), and water uptake (1149%) - The scaffold promoted proliferation of human dermal fibroblasts for wound healing	Esparza et al. (2018)
Lipids	Plasticizer (Bio-epoxy)	- Lipids were extracted using supercritical CO_2_ - Bio-epoxy was produced by epoxidation of the extracted lipids	- Spent hen lipid-derived bio-epoxy materials were produced	Safder et al. (2019a, b)
Lipids	Polymer precursor (ethenolysis)	- Ethenolysis was achieved by a microwave-assisted solvent-free approach with catalysts	- Spent hen lipids as a renewable lipidic source for ethenolysis	Pradhan et al. (2020)
Lipids	Bionanocomposite film	- Compression molding using spent hen-derived fatty acids and nanoclay	- Produced a lipid-based bionanocomposite film with enhanced thermal stability and flame retardancy compared to neat homopolymer	Safder et al. (2020)
Feather	Adsorbent	- Feather was functionalized with silver nanoparticles	- Enhanced adsorption capacity of rhodamine B by 30 folds	Azeez et al. (2020)
Feather	Adsorbent	- Treatment with hydrogen peroxide	- Effectively removed hazardous acid dye Amido Black 10B from aqueous solutions	Mittal et al. (2013)

#### Myofibrillar proteins and collagen (wood adhesive, bio-plastic, and hydrogel)

Wang and Wu (2012) reported for the first time the preparation of spent hen proteins-based wood adhesives. Crude spent hen muscle protein extract (mostly myofibrillar proteins) was modified by sodium dodecyl sulfate (SDS) (0.5–5%) or urea (1–8 M), with the optimized incorporation rates as 3% SDS and 3 M urea. Use of either modification agent enhanced protein unfolding, exposing more secondary structures that interact with wood substances, thus strengthening protein-wood bonding. The prepared adhesives were applicable in both dry and wet environments. Myofibrillar proteins have also been used to prepare bionanocomposite films for food packaging application, with the addition of glycerol as the plasticizer, chitosan as the cross-linker, and nanoclay as the nano-reinforcement; the product possessed satisfactory thermal, thermomechanical, and barrier properties (Zubair et al. 2019). Likewise, spent hen collagen has been used to prepare hydrogels for tissue engineering, which promoted the proliferation of human dermal fibroblasts, demonstrating a potential wound healing application (Esparza et al. 2018).

#### Lipids (plasticizer, polymer precursor, and bionanocomposite)

Spent hen has a high content of lipids (~ 15%, wet basis, Table 1), representing another major component being utilized as biomaterials. For example, fatty acid methyl esters prepared by transesterification of spent hen-derived triglycerides were used to generate linear α-olefins by ethenolysis, the raw materials for synthesizing polyethylene, oxo alcohols, and poly-α-olefins (Chatterjee and Jensen 2017; Pradhan et al. 2020). Safder et al. (2019b) extracted lipids from spent hens followed by converting them into bio-epoxy by epoxidation for bio-plasticizer production. The same researchers also prepared bionanocomposites using the lipids and nanoclay, which exhibited higher thermal stability and flame retardancy than those of neat homopolymer (Safder et al. 2020).

#### Feather/keratin (adsorbents)

Feather generally accounts for about 5–7% of bird body weight, consisting of 90–92% proteins, mostly keratin. Keratin is highly specialized fibrous proteins and is insoluble in water, organic solvents, weak acids and alkalis. It is also resistant to proteolytic degradation of common enzymes such as pepsin and trypsin due to the abundance of hydrogen bonds, salt linkages, and disulfide linkages; only disrupting these interactions allows keratin to swell and expose amino acids and makes its extraction feasible (Nakamura et al. 2002). Feather keratin is mainly processed by chemical (e.g., strong acids or alkalis, oxidizing or reducing agents) or biological (e.g., microbial and enzymatic hydrolysis) treatment, before being utilized. Hen feathers have been developed as wastewater adsorbents. Azeez et al. (2020) prepared an adsorbent using hen feather functionalized with silver nanoparticle, which enhanced the adsorption capacity of rhodamine B by 30 folds; rhodamine B is a synthetic cationic dye with neurotoxicity and genotoxicity and can cause hormonal disturbances and irritation of skin, eyes, respiratory tract, being used widely in the production of textiles, paper, drinks, foods, and leathers. Mittal et al. (2013) developed hen feather as adsorbent for adsorption of another industrial dye, Amido Black 10B. More earlier reports on using hen feather for wastewater treatment can be found in Naushad and ALOthman (2015). Direct evidence on hen feather-based biomaterials is limited, due to lack of details on sources of feathers, however, feather keratin has been used for biofuel production as well as biomaterial and biomedical applications such as wood adhesives, bioplastics, and hydrogels, among others (Esparza et al. 2018; Zahara et al. 2021).

## Conclusions and perspectives

**Fig. 3:**
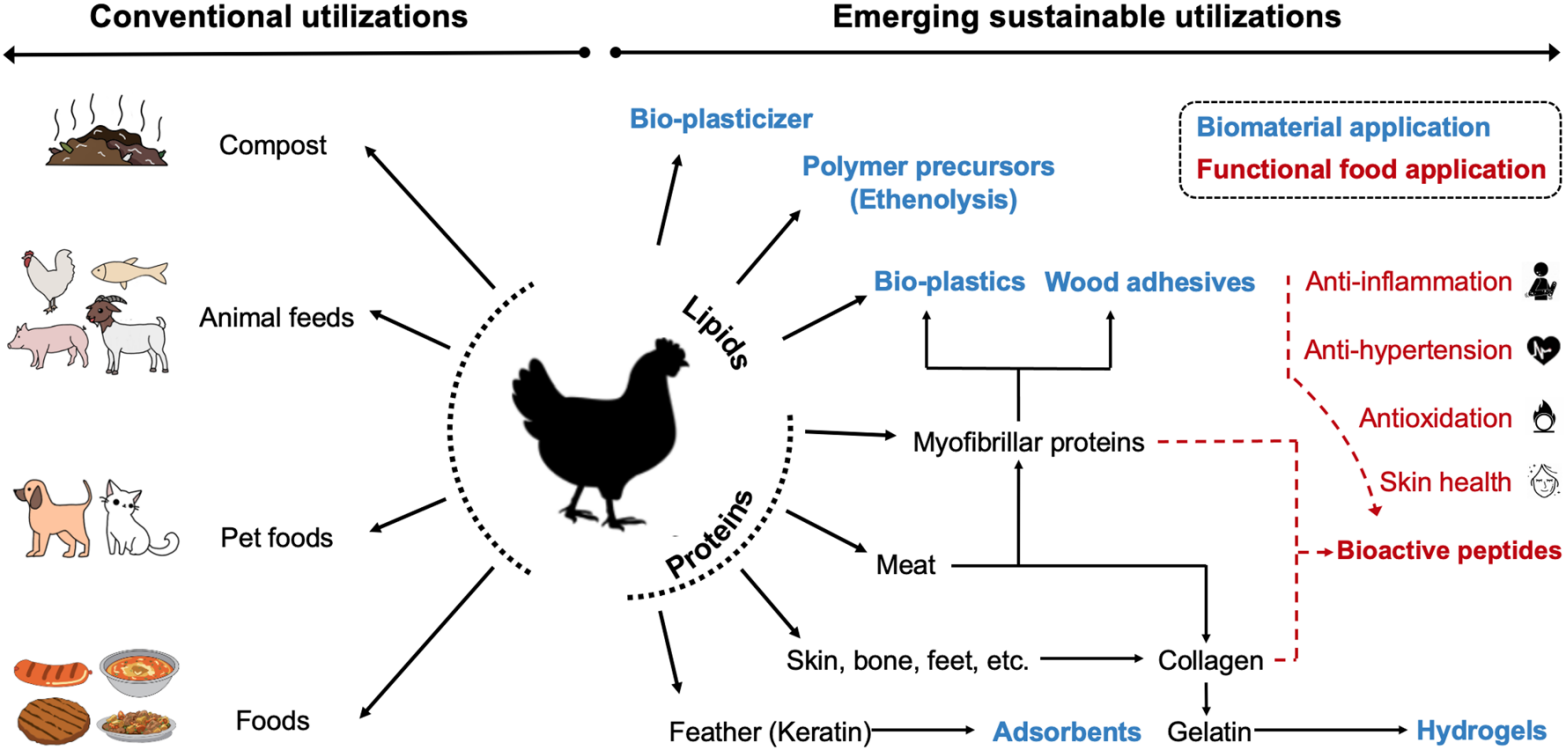
Overview of conventional and valorized uses of spent hens

Every year, billions of spent hens are produced globally in the egg industry. Fundamental differences in conceptualizing spent hens as food products among countries shape different strategies in disposing and processing spent hens. In Asia, spent hens are popular in Chinese cuisine and are available as various meat products or snacks in India; while in the western society, they are considered as an egg-industry waste, not for food use, and are instead primarily disposed by landfilling or being rendered into animal feeds or pet foods. Emerging sustainable utilizations are being explored for valorized uses of spent hens, including functional food ingredients (e.g., gelatin, LMW collagen peptides, bioactive peptides), and protein/lipid-based polymerized biomaterials (e.g., adhesives, bioplastics, bionanocomposites, hydrogels, adsorbents, etc.); future directions will be likely to continue to center on these two fields of research. Both conventional and emerging utilizations of spent hens are illustrated in Fig. 3.

Currently, both functional food ingredients and biomaterials derived from spent hens are based on purified protein or fat. Thus, it is imperative to develop a simple but multifunctional protocol, which enables separation and extraction of fat and various proteins simultaneously or sequentially. This can significantly reduce the cost of converting spent hen biomass into end products, thus facilitating their future industrial applications. For example, enzymatic hydrolysis using ground whole spent hen carcasses enabled the separation of protein and fat, yielding the protein fraction suitable for human consumption and oil fraction being of good quality (Hjellnes et al. 2020). Besides, spent hen is rich in collagen with high crosslinking degree (Hong et al. 2019). Hence, new methods, such as hydrothermal processing and subcritical water hydrolysis, should be explored to break down the highly crosslinked collagen before being developed for food and cosmetic uses (Adams et al. 2018; Dong et al. 2014; Melgosa et al. 2021).

Not all the byproducts of spent hens have been discussed in depth due to lack of studies in the literature. The offal waste including heads, feet, viscera, and blood accounts for ~ 30–40% of the living body weight (Lasekan et al. 2013). Heads, feet, and viscera contain 11–16% of proteins; blood meal contains 60–80% of proteins. They can be developed as protein meal or hydrolyzed collagen (gelatin), or be enzymatically hydrolyzed as polypeptides with excellent functional, nutritional, and health-beneficial benefits (Lasekan et al. 2013); viscera has a high fat content which is a good source of animal fat (Peña-Saldarriaga et al. 2020). Besides, combs and wattles are suitable materials for the extraction of glycosaminoglycans such as hyaluronic acid (Abdallah et al. 2020). Feather has a protein content as high as of 90%, mainly composed of keratin. Through physical, chemical, and biological treatments, feather proteins can be degraded as fertilizers, animal feeds, or bioactive peptides, or be extracted and developed for biodiesel, biomaterial, and biomedical applications (Karuppannan et al. 2021).

Our review shows the promising valorized products of spent hens through various physical, chemical, or microbiological treatments. However, it should be noted that there is a lack of consensus in the technical approaches from spent hen pretreatments to processing conditions, e.g., different starting portions of spent hen carcass, different biomass extraction approaches, etc. Hence, quantitative data regarding the conversion from raw spent hen carcasses to the final products have only been preliminary discussed. Product positioning is also important, e.g., fat as animal feed additives or for human assumption. This review is the first work summarizing the previous and current research status of spent hen uses, and will prompt the development of more uniform and widely accepted approaches in the valorized uses of spent hens.

Despite being a waste in the poultry and food sectors in many western countries, spent hen is commonly consumed in other cultures like some Asian and African countries. Hence, a potential bridge may be established for exporting raw spent hen carcasses or products such as stewed chicken and soups from the supply side to the demand side. Indeed, many underdeveloped countries face various degrees of hunger, malnutrition, and food insecurity as well as lack of intake of high-quality proteins. Processing spent hens for food uses, other than being disposed by landfilling or as feedstuffs or pet foods, is more sustainable and environmental-friendly; it also increases food availability, as called on by the United Nations Sustainable Development Goals 2030.

## Data Availability

All data/materials are included in the article.

## Abbreviations

ABTS: 2,2ʹ-Azino-bis(3-ethylbenzothiazoline-6-sulfonic acid)
ACE: Angiotensin-converting enzyme
ACEi: Angiotensin-converting enzyme inhibitory
ACE2: Angiotensin-converting enzyme 2
ACE2u: Angiotensin-converting enzyme 2 upregulating
AGEs: Advanced glycation end products
DHE: Dihydroethidium
DPPH: 2,2-Diphenyl-1-picrylhydrazyl
FICA: Ferrous ion chelating activity
FRAP: Ferric reducing antioxidant power
ICAM-1: Intracellular adhesion molecule-1
IL: Interleukin
LMW: Low-molecular-weight
MMP: Metalloproteinase
SDS: Sodium dodecyl sulfate
TNFα: Tumor necrosis factor alpha
VCAM-1: Vascular cell adhesion molecule-1
